# CEVAP Journal: the first Brazilian electronic scientific publication turns 20 years old

**DOI:** 10.1186/s40409-015-0050-7

**Published:** 2015-12-08

**Authors:** Benedito Barraviera

**Affiliations:** CEVAP/UNESP, Caixa Postal 577, Fazenda Experimental Lageado, Rua José Barbosa de Barros, 1780, 18610-307 Botucatu, SP Brasil

The year 2015 marks 350 years since the appearance of the world’s first scientific publication and 20 years since the publication of the *Journal of Venomous Animals and Toxins including Tropical Diseases* (JVATiTD – www.jvat.org) [1–3]. It was the first Brazilian electronic scientific publication – an official journal of the Center for the Study of Venoms and Venomous Animals (CEVAP) of São Paulo State University (UNESP) [4].

The printing-press revolution, one of the most important breakthroughs for humanity, began about 1465 when Johannes Gutenberg invented a moveable mechanical type for printing. In 1665, the first scientific journals appeared in France and England, called respectively the *Journal des Sçavans* and *Philosophical Transactions*. Since then, the structure of a scientific work has respected the basic paradigms “introduction, methods, results and discussion” – one of humanity’s oldest formats.

In 1900, 9,000 scientific articles were published per year; by 2000, this number had grown to 900,000. In 2010, PubMed Central, which does not index the majority of the world’s journals, received 1,100,000 papers for indexing, i.e., approximately 3,000 per day [5]. These data demonstrate the exponential growth of the world scientific knowledge. The great challenge was: how could this immense amount of knowledge be printed and delivered by mail?

In August of 1991, Paul Ginsparg, a physicist at Cornell University (USA), founded the Los Alamos e-print archives (http://arxiv.org/), which was considered the first electronic scientific publication [6]. Its mission was to distribute papers automatically to cover the areas of physics, mathematics, computer science, quantitative biology and statistics. It was an electronic system of automatic distribution with definitive submission or the possibility of a correction by the author. It was also discussed in the 1990s whether an electronic publication should be considered a scientific journal or not. According to the Vancouver Group, any electronic publication made available on the Internet constitutes a publication [7].

In 1995, there were about 100 electronic journals in the world, and the commercial Internet was starting in Brazil. In this context, the first Brazilian electronic scientific journal was launched in Botucatu, covering the area of Toxinology. This new journal started one hundred years after Vital Brazil lived in Botucatu. It is a homage we paid to this brilliant Brazilian scientist [4]. *The Journal of Venomous Animals and Toxins* (JVAT) circulated until 2002. Between 1995 and 1999, it was distributed on floppy disks, between 2000 and 2002 on CD-ROM, and only from 2003 on by the Internet, initially via the website http://www.jvat.org.br. In the same year, the publication was broadened to include tropical diseases, and was then called the *Journal of Venomous Animals and Toxins including Tropical Diseases* (JVATiTD) [8].

In 2006, the journal was selected to be included in the Science Citation Index Expanded (ISI – Web of Science™ – Thomson Reuters) and in 2007, received its first impact factor, 0.43. Since between 2007 and 2012 the impact factor remained steady between 0.3 and 0.5, the Editorial Board decided to migrate to a renowned international publisher that would maintain the open access policy and contribute to improving the quality of the publication. It was decided at the time by BioMed Central, Springer. After initiating the partnership, the following goals were established: to index immediately in PubMed Central; to maintain the policy that APCs (article-processing charges) would be free to UNESP researchers; to restructure the editorial board by inviting new researchers, preferentially foreigners; and to increase the impact factor to 2.0 within five years.

In January of 2013 there was a migration to BioMed Central and indexing in the following databases: PubMed, PubMed Central, PubMed Central Canada, UK PubMed Central, Depot (National Library of the Netherlands), SpringerLink, Springer Images/Author Mapper, CrossRef, Elsevier/ELSAMS, Quertle, Scirus, EPO (European Patent Office), OhioLINK, SIMID, CISTI (Canada Institute for STI), Proquest, GALE and WebCite/Webcitation. Five associate editors were invited and the editorial board than comprised 15 Brazilian members and 13 foreigners (three from France and the others were from Cuba, Egypt, China, Venezuela, Argentina, Nigeria, the United Kingdom, Mexico, Turkey and Poland). In 2015, the impact factor was 0.8, demonstrating the growth of the publication.

A study conducted by Eysenbach [9] showed that the articles published in open access were cited twice as often the paid ones four to ten months after publication. An electronic open-access journal not only provides higher velocity and visibility, but consequently its articles are cited more frequently, thereby improving its impact factor.

Barraviera [10] lists the main reasons for publishing in open-access electronic journals: they support all types of images with no size limit; they support different media (audio and video); continuous publication increases the availability on the Internet; indexing in different search engines; accessibility and support to different platforms (computers, tablets, smartphones among others); greater visibility – and therefore a bigger chance of citation and finally an increased impact factor (Additional file 1).

## Conclusion

The creation of an electronic scientific journal 20 years ago, when the commercial Internet was not available in Brazil, was a truly daring act! This paradigm shift could come true because it was developed step by step, i.e., first the floppy disk attached inside the cover with the possibility of printing, next the CD-ROM enabling reading on a computer screen, and then the availability of content and submission of papers only online. The electronic format itself became a reality in 2013, when the journal moved to the continuous publication model. Our story clearly shows that some paradigm shifts must occur gradually so that success can be achieved.

## Additional file

Additional file 1:
**Video “JVATiTD – 350 Years of Scientific Publishing: from Gutenberg to smartphones”.** Available at: https://www.youtube.com/watch?v=lhdP3P4BrRU. (MP4 32.9 mb)
